# 316L Stainless Steel Powders for Additive Manufacturing: Relationships of Powder Rheology, Size, Size Distribution to Part Properties

**DOI:** 10.3390/ma13235537

**Published:** 2020-12-04

**Authors:** Robert Groarke, Cyril Danilenkoff, Sara Karam, Eanna McCarthy, Bastien Michel, Andre Mussatto, John Sloane, Aidan O’ Neill, Ramesh Raghavendra, Dermot Brabazon

**Affiliations:** 1 Advanced Processing Technology Research Centre, Dublin City University, Collins Avenue, 9 Dublin, Ireland; eanna.mccarthy@dcu.ie (E.M.); bastien.michel@grenoble-inp.fr (B.M.); andre.mussatto2@mail.dcu.ie (A.M.); dermot.brabazon@dcu.ie (D.B.); 2 I-Form Advanced Processing Technology Research Centre, Dublin City University, Collins Avenue, 9 Dublin, Ireland; RRaghavendra@wit.ie; 3 South East Applied Materials (SEAM) Research Centre, Applied Technology Building, Waterford Institute of Technology, X91 TX03 Waterford, Ireland; cyrilkof@outlook.com (C.D.); SKaram@wit.ie (S.K.); 4 Particular Sciences, 2 Birch House, Rosemount Business Park, Ballycoolin Road, 11 T327 Dublin, Ireland; johnsloane@particular.ie; 5 Castolin Eutectic, Magna Business Park, 36 Magna Avenue, Citywest, 24 Dublin, Ireland; aidan.oneill@castolin.com

**Keywords:** metal additive manufacturing, powder bed fusion, powder rheology, microstructure, Stainless Steel 316L

## Abstract

Laser-Powder Bed Fusion (L-PBF) of metallic parts is a highly multivariate process. An understanding of powder feedstock properties is critical to ensure part quality. In this paper, a detailed examination of two commercial stainless steel 316L powders produced using the gas atomization process is presented. In particular, the effects of the powder properties (particle size and shape) on the powder rheology were examined. The results presented suggest that the powder properties strongly influence the powder rheology and are important factors in the selection of suitable powder for use in an additive manufacturing (AM) process. Both of the powders exhibited a strong correlation between the particle size and shape parameters and the powder rheology. Optical microscope images of melt pools of parts printed using the powders in an L-PBF machine are presented, which demonstrated further the significance of the powder morphology parameters on resulting part microstructures.

## 1. Introduction

The worldwide success of additive manufacturing (AM) is thanks to the exceptional opportunity to produce complex near-net-shapes in a single process. By adding material layer upon layer, a pre-programmed three-dimensional (3D) model is formed without the extensive subtracting methods associated with conventional production. The key to the success of AM will be in understanding the relationship between process variables, material properties and final structure. Metal based additive manufacturing has received increasing attention in recent years, both from academic researchers and from industries such as aviation [1,2,3,4,5] and the medical devices sectors [6,7,8,9,10]. In the last two years alone, the number of metal AM machines and powder suppliers have increased dramatically [11].

The increased usage and demand for metal AM has led to an increase in the number of investigations into the complex, multi-variate relationships between the build parameters used and the resulting part properties. Materials such as Nitinol, 316L stainless steel, nickel super-alloys 625 and 718, Al alloys 6061, 7075, reinforced composites and Ti6Al4V are of interest for a variety of industries [12,13,14,15,16,17,18,19,20,21,22,23,24,25,26,27,28,29]. 316L stainless steel has good ductility, strength, temperature resistance, biocompatibility, corrosion and fatigue resistance, cost and availability [30,31,32,33]. Due to these desirable properties, it is used in a wide variety of commercial applications, from aerospace to medical devices, as well as household items and industrial vessels. From this analysis of the literature, it has been found that part density, surface finish and mechanical properties are strongly influenced by the process parameters employed, including the powder properties. A deeper understanding of how the powder flows and how morphological properties affect the finished parts is important, in order to develop a full, robust process understanding for the production of high-quality parts. This understanding is required in order to improve produced part density, powder re-usability and powder transit through the printer itself. In recent years, several groups have reported on the interdependent nature of powder properties, the effect of powder properties on part characteristics and the variation of the powder properties with repeated use in the AM process [24,25,29,34,35,36,37,38]. The topic of powder spreadability has been increasingly investigated in the context of AM processes. These investigations are based on the notion that in a laser-powder bed fusion (L-PBF) machine, the powder *spreading* characteristics are at least as important as its more traditional rheological (or flow) properties [24,37,39,40,41,42]. Particle shape is considered important as an irregular shape can lead to increased friction between particles. Hausnerova et al. [25] found however that the effect was more complicated, that the powder flow energy or torque measured was also dependant on particle size and to a lesser extent whether the powder was produced using a gas or water atomization process. Others have found that a powder with a large amount of particles below 10 µm increased cohesive forces and resulted in the powder being unsuitable for the PBF process, due to poor rheological performance [38]. To add to this body of knowledge, this paper presents a new detailed examination of the complex relationship between powder shape, size, composition and resultant powder fluidity with microstructure and surface roughness of parts produced using two different 316L powders.

In this paper, the properties of two inert gas atomised 316L stainless steel powders are presented. The flowability, particle size and particle shape were measured and analysed for both powders. The correlation between the particle size and shape and the powder rheology was investigated, and the suitability of the experimental powder for use in AM was determined, based on the microstructure examination of parts produced with these powders, tensile properties and surface roughness, an indication of surface finish.

## 2. Materials and Methods

Two different 316L powders were investigated in this work, referred to as Powder A and B. Both were produced by the inert gas atomisation process.

**Figure 1:**
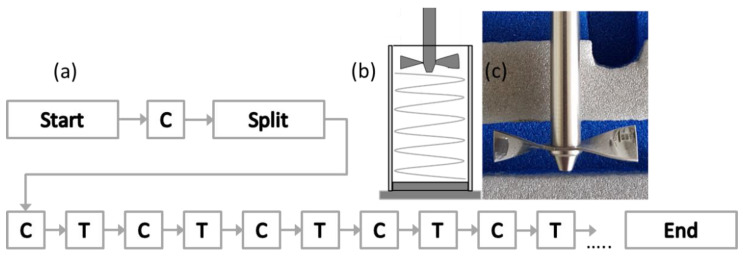
Fluidity test methods for (**a**) the stability test sequence (C = conditioning cycle, T = test cycle), (**b**) schematic representation of the blade motion, both adapted from [43] and (**c**) photograph of the 23.5 mm testing blade.

The powder rheology studies were performed using a Freeman Technology FT4 Powder Flow Analyser (Freeman Technology, Tewkesbury, UK). For all powder tests performed with the FT4, a glass vessel of 25 mL volume was used (the stability test, test sequence shown in Figure 1a), with either a 23.5 mm stainless blade (see Figure 1b,c).

The vessel (Figure 1b) is split at the beginning of the sequence to provide a precise volume of powder for measurement. The Basic Flowability Energy (*BFE*) (Equation (1)) is determined during the downward motion of the blade (confined regime), while the Specific Energy (*SE*) (Equation (2)) is calculated when the blade moves upwards through the powder (unconfined regime). The BFE value was taken from energy test number 7; as after six conditioning tests, the powder is considered to be homogeneous and conditioned, meaning the effect of powder handling has been negated. The Stability Index (*SI*) (Equation (3)) is also calculated in this test and is an indication of the stability of the powder during the test. Figure 1c shows an illustration of the blade geometry. Error bars are based on a 95% Confidence Interval (CI).

(1) BFE=Energy Test 7 (mJ)

(2) $$SE=\;\frac{\frac{\left(Up\;Energy\;Cycle\;6\;+\;Up\;Energy\;Cycle\;7\right)}{2}}{Split\;Mass}\;\left(mJ/g\right)$$

(3) $$SI=\;\frac{Energy\;Test\;7}{Energy\;Test\;1}$$

In the compressibility test, the variation of the powder density is calculated as a function of increasing applied normal stress. Compressibility is directly influenced by many factors such as particle size distribution, shape, texture and cohesivity [44].

During the aeration test, a continuous regulated stream of air is introduced from the bottom of the powder vessel, at velocities varying from 0–10 mm/s. The test measures the variation in flow energy as a function of decreasing air velocity.

**Figure 2:**
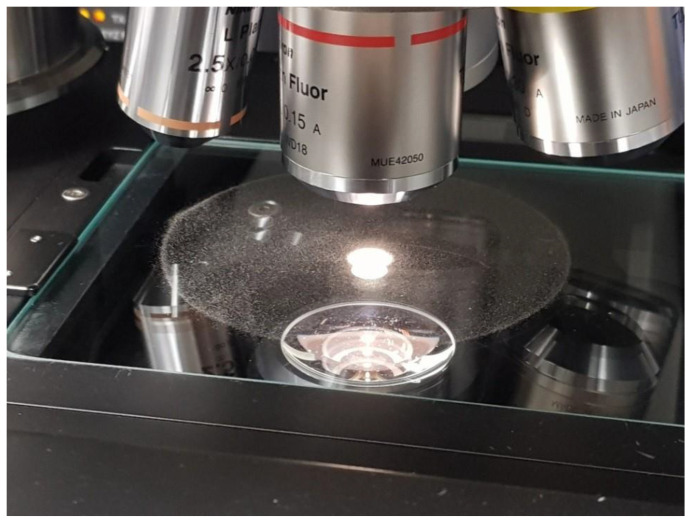
Image of dispersed powder test method for analysis of particle shape.

Particle size analysis on the powder samples was performed using a Malvern Mastersizer 3000 (Malvern Panalytical UK, Malvern, UK), fitted with an Aero S solid dispersion unit (Malvern Panalytical UK, Malvern, UK) and a stainless-steel venturi hopper plate dispenser (Malvern Panalytical UK, Malvern, UK). Pressure was maintained in the range 0.5–1 bar, and feed rate was varied between 7–20%. Occlusion values were maintained in the range of 0.5–6%. All powder samples were thoroughly mixed by manually rotating the sample container top-over-bottom 30 times before withdrawing the powder aliquot required for the test. Data were recorded in terms of the % volume distribution against particle size using a spherical model approximation. In all cases, an average and standard deviation were calculated. The shape and morphological properties of the powder particles were investigated using a Malvern Morphologi G3 (Malvern Panalytical UK, Malvern, UK). For this, a 3 mm^3^ volume of metal powder was dispersed under 1 bar pressure onto a glass slide using compressed air. These volume and pressure settings were found to give an acceptable dispersion, where particles were sufficiently well separated from each other to enable identification and measurement of individual particles using the image recognition software in the Morphologi G3 (Morphologi Version 8.23, Malvern Panalytical UK, Malvern, UK). Where particles were irregular or appeared to overlap, they were either discounted from the subsequent analysis or further in situ analysis using variable focus stacking was conducted to distinguish between overlapping and irregularly shaped particles. Four scan areas were chosen, and the instrument identified between 100,000 and 400,000 particles in each sample, see Figure 2 for experimental setup. The circularity was measured as defined in Equation (4):

(4) $$\mathrm{Circularity}\;=\;\frac{4\cdot \pi \cdot A}{P^{2}}$$

where *A* is the area of the particle, and *P* is the perimeter of the particle.

Compositional analysis of the powder samples was obtained using a Hitachi S-3000N VP Scanning Electron Microscopy (Hitachi HHT UK and Ireland) with integrated Energy Dispersive X-ray Spectrometer (SEM-EDX) (Hitachi HHT UK and Ireland).

The powders were processed in an EOS M280 L-PBF machine (EOS GmbH, Krailling, Germany). Table 1 outlines the process parameters used. The parts produced were then investigated for surface roughness and tensile properties. Dog-bone shaped test samples were fabricated in X, Y and Z build orientations for tensile testing, in accordance with ASTM E8/E8M [45]. The ratios of the dimensions of the dog bone test samples were reduced while still remaining in adherence to the dimensions stipulated in E8. This was done in order to allow for the Z oriented samples to be produced with the powder supply available in the EOS machine powder reservoir and the supply factor selected for the samples.

The tensile properties were characterized using a Zwick 50 kN tensile testing machine Zwick Ltd., Leminster, UK) and Epsilon extensometer (Zwick Ltd., Leminster, UK). Experiments were controlled and results analysed using Zwick TestXpert software (Zwick Ltd., Leminster, UK). Stress-strain curves for each sample were obtained, and from these, the elastic modulus, ultimate tensile strength (UTS) and extension reached before breaking determined.

The surface roughness, R_a_, was measured using a Bruker-Contour GT white-light interferometer (Bruker UK Ltd., Coventry, UK) with a magnification of ×27.5 and a measurement area of 230 × 172 µm, averaging three measurements for each location before and after peening.

A more detailed discussion of the L-PBF work is to be found in a separate publication currently under preparation.

## 3. Results

Powders can be considered to be a mix of solid (the particle itself), liquid (on the powder surface) and gas (air or another gas entrained between the powder particles). Their flow properties are therefore highly complex and inter-dependent. Consequently, the interpretation of powder properties cannot be achieved from a single test, or analytical technique. Therefore, the approach of this work was to investigate rheology, size, shape and composition of the powder to more fully understand the effects that these properties may have on parts fabricated using metal additive manufacturing. The Pearson correlation method was used to identify correlations between parameters of interest.

### 3.1. Investigation of Powder Rheology

A mix of bulk and dynamic tests were utilised to characterize the rheological properties of the powders investigated. The FT4 instrument used is capable of 16 different tests; however, three tests were considered to be the most relevant to this work and for the use of a powder in L-PBF additive manufacturing process.

**Figure 3:**
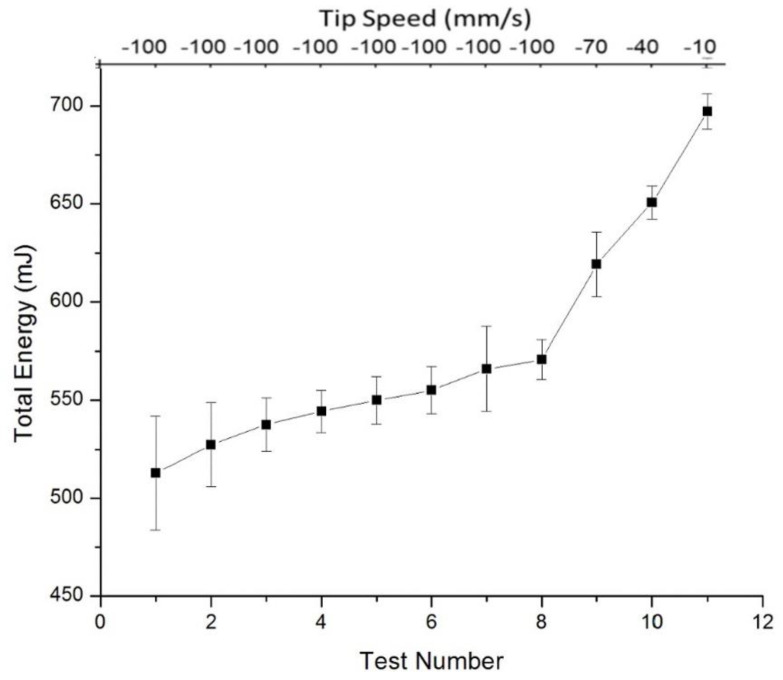
The results of eleven Variable Flow Rate (VFR) and Stability fluidity tests for Powder A are shown with the total energy noted versus the test number and tip speed. The negative sign for tip speed is a convention denoted tip direction. Three separate, random test samples of Powder A were analysed. The three runs were averaged and 95% CI bars are shown.

**Figure 4:**
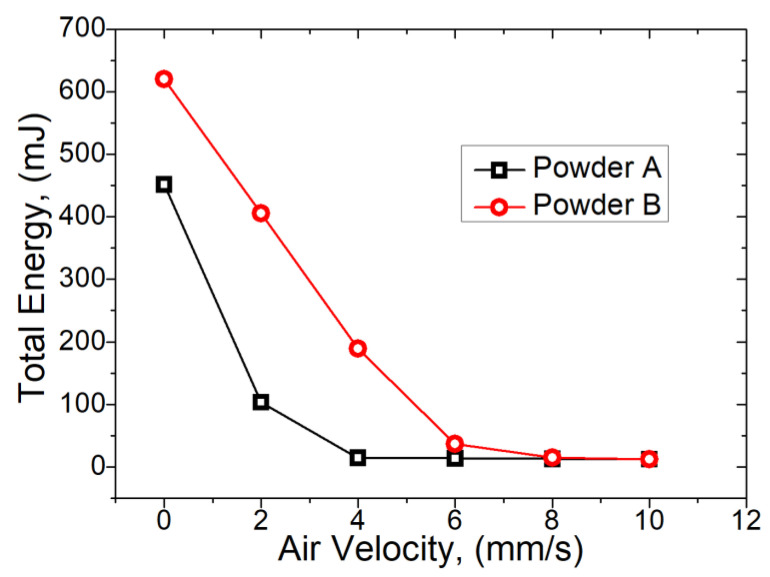
Comparison of fluidization characteristics for Powder A (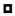) and Powder B (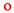).

**Figure 5:**
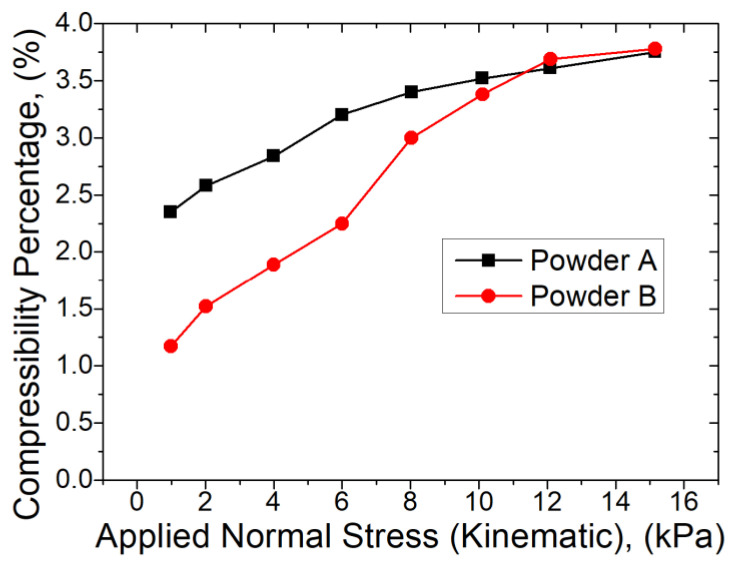
Comparison of compressibility characteristics exhibited by Powders A (■) and B (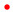).

**Figure 6:**
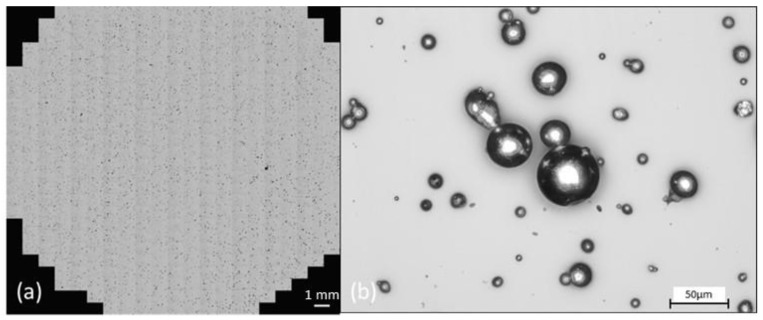
Powder shape analysis micrographs showing (**a**) a 10.8 mm radius scan area and (**b**) magnified view of the particles.

The results of the different rheology tests are presented. The results from the three (stability, compressibility and aeration) tests performed on Powders A and B are shown in Figure 3, Figure 4, Figure 5 and Figure 6. Figure 3 shows the results from the Variable Flow Rate (VFR) and stability test measurement runs. The lines represent the results for three separate runs for Powder A. The first seven tests are the stability tests, and tests 8 to 11 show the results for the VFR measurements. The results from three virgin aliquots of Powder A are shown in the three series. After the seventh run in each series, the powder was considered to be conditioned whereby the total energy measured plateaued and the BFE was therefore measured in test 7. The blade tip speed was 100 mm/s for the first eight tests and was reduced to 70, 40 and 10 mm/s for the VFR tests 9, 10 and 11 respectively.

From Figure 3, a first preliminary interpretation of the data can be obtained by looking at the curve’s shapes. The BFE is seen to plateau around Tests 6–7, which is to be expected after the repeated conditioning cycles. A linear increase is observed in Tests 8–11, which is consistent with the reduction in tip speed in these tests, as the blade rotates more slowly, a higher torque force is experienced from the powder particles in the confined regime. As noted earlier, the BFE value is taken from Test 7 for each aliquot of powder. BFE/g is a common metric for powder flowability and the BFE/g was found to be 4.46 ± 0.12 mJ/g for Powder A and 6.36 ± 0.21 mJ/g for Powder B.

The analysis of the basic flowability energy is more complex, mostly because, as previously stated, this metric is dependent on a number of interrelating powder properties. To fully evaluate flow behaviour, it is useful to study this parameter and others and compare such values for different powders. Analysis of the SE values of 1.84 (Powder A) and 2.58 (Powder B), suggests that both powders can be considered to possess low cohesion, however powder B has a value almost 50% higher, indicating that cohesion is more evident. Values below 5 are considered to demonstrate low cohesion. The SE value is calculated while the powder is in an unconfined regime and is less dependent on compressibility effects than the BFE.

The results of the aeration test are shown in Figure 4, in which the torque experienced by the blade rotating within the powder is evaluated at different air velocities. Powder A and B exhibit markedly different flow behaviours at lower air velocities and by analysing the results, it can be seen that powder A has a value of minimum fluidisation velocity at 4.00 mm/s with powder B exhibiting complete fluidisation at 8.00 mm/s. At higher air velocities (>6 mm/s), both exhibit almost zero flow energy and exhibit AE_10 values of <10 mJ at air velocities >8 mm/s.

The compressibility test results are shown in Figure 5. The split mass of the powder is the same as that used in the Stability and VFR tests.

In Figure 5, there is an increase in percentage compressibility between approximately 1–10 kPa before plateauing above 10 kPa for both Powder A and B. For Powder A, percentage compressibility at the initial applied stress is approximately 2.3%, increasing to around 3.75% at maximum load. Powder B shows similar behaviour, but its initial percentage compressibility is lower, around 1.2%. It is inferred that these values are approximately the critical stress values at which on average satellite particles break free from the primary particles. That would explain the graph trend, because, by separating primary particles and satellites, the powder components would become more spherical, hence able to pack more efficiently and therefore a lower compressibility. The presence of fine satellite particles would also aid in improving packing efficiency. However, both powders generally exhibit low levels of compressibility, as would be expected of highly dense, well packed metal powders. Some AM machines use a compression step in each powder recoating process, therefore, these results are relevant for such processes, more work could be done to ensure that the stress range across a similar cross sectional area to that examined in this test is similar to that used in AM machines.

### 3.2. Investigation of Particle Size and Shape

In order to gain a deeper understanding of the properties of the metal powders under investigation, a quantitative evaluation of the shape and size of the particles is required. Traditionally, shape analysis is performed by dispersing a powder on to a substrate and acquiring an optical microscope image. This image is then usually processed using image analysis software such as ImageJ™. While this process yields good quality data, it can suffer from slow processing times and subjective interpretation of particle shapes, grayscale mask generation and image lighting issues. A different approach utilising the Morphologi G3 instrument was adopted here. This allowed for a controlled dispersion of powder and a systematic imaging of the dispersion, followed by image stitching of the various image files. In this way, very large numbers of powder particles can be dispersed with suitable distances between the particles to allow for identification of shape and morphology characteristics of individual particles. All particles were imaged and could be individually analysed if needed. The morphological data presented here represent (Table 2) an average of each morphological parameter across the global particle population, with larger (non-powder) entities removed from the analysis. Figure 6 shows as visual microscope image of a global view of the scan area of Powder A dispersion used in particle shape analysis and (b) a zoomed-in view of the particles. Scale bar indicates particles are of the order of 20–50 μm.

As Table 2 illustrates, powder A contains a higher number of fine particles (<10 μm) than powder B. This is confirmed in the D_10_ values for each powder and in the similar D_50_ and D_90_ values for each powder. The presence of fines does not appear to adversely affect the rheological properties however, as Powder A exhibits better flow properties and is more easily fluidized in this flow regime. Satellite particles are observed in the shape analysis optical microscope images (Figure 6).

**Figure 7:**
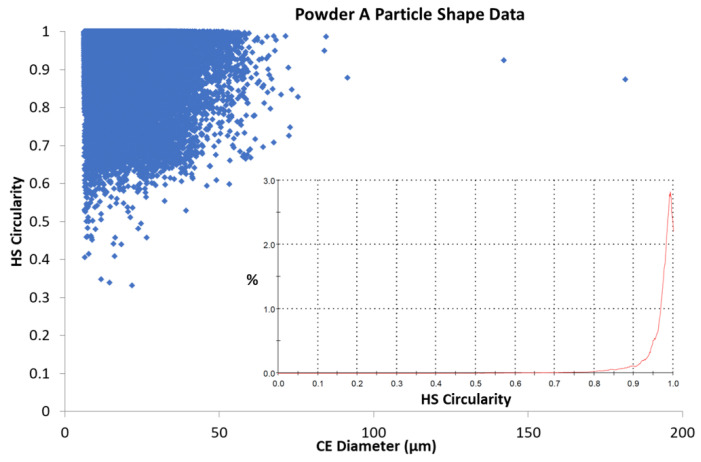
Relationship between particle circularity and Circular Equivalent (CE) Diameter for powder A. (Inset): Number based distribution for HS Circularity.

**Figure 8:**
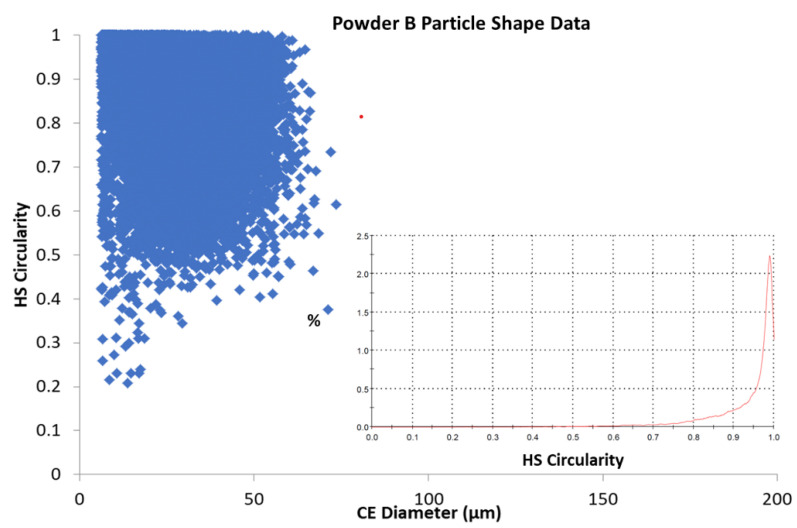
Relationship between particle circularity and Circular Equivalent (CE) Diameter for Powder B. (Inset: Number based distribution for Circularity.).

Figure 7 and Figure 8 illustrate the relationship between the calculated circularity or sphericity of particles from Powder A and Powder B and their calculated diameters. A higher degree of sphericity was observed for Powder A particles across the 0–50 μm range than that found for Powder B particles in a similar particle size range. Lower Circularity values were observed for smaller particles in both powders; this could be attributed to the lower resolution for smaller particles and hence a lower number of pixels for these particle images.

As noted, both powders exhibit high circularity across their respective particle size distributions, shown in Figure 7 and Figure 8, which illustrate a scatter plot of each particle’s circularly equivalent (CE) diameter with its calculated Circularity (Equation (1)). The scatter plots demonstrate that both powders have Circularity values approaching 1 and are therefore considered to be highly spherical. Powder B appears to have more irregularly shaped particles (High Sensitivity (HS) values < 0.4) than Powder A has, and this is more noticeable for the smaller particles (<20 µm). The inset graphs in each figure show smoothed graphs of the data for each of the possible HS Circularity gradations (0–1.0) and these correlate with the parent data, a longer tail is seen for Powder B around 0.8–0.9 and this is taken to indicate that this powder, while quite spherical, has marginally lower sphericity (0.94) than that of Powder A (0.97), based on the particles measured for each sample. It is important to point out that approx. 128,000 particles of Powder A were analysed and used for the calculations, while a smaller sample size (some 28,000) particles of Powder B were analysed. It is possible that this may account for some of the differences observed, it could be assumed that given the similar Laser Diffraction Scattering (LDS) measurements, a larger number of particles would mean a greater number of fines in the sample and this is in agreement with the LDS results. Therefore, the experimental sample still has a greater sphericity value globally. Table 3 shows the relevant calculated values for the powders.

The average aspect ratio for Powder A was 1.05 times greater than the aspect ratio calculated for the commercial powder. This may be due to a greater number of fines in Powder A and which could also be more spherical than those in the corresponding size range of Powder B.

### 3.3. Powder Surface Morphology and Composition

**Figure 9:**
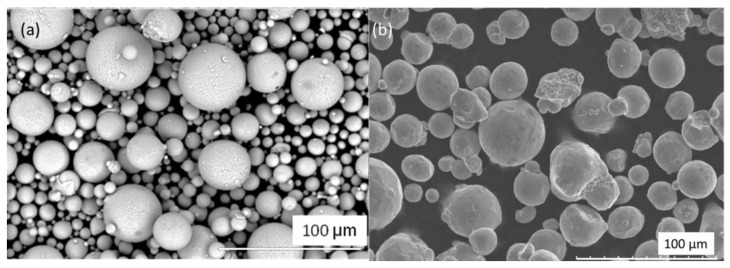
Captured micrographs of (**a**) backscatter detection from Powder A and (**b**) secondary electron detection from Powder B, both images were taken at 20.0 kV.

**Figure 10:**
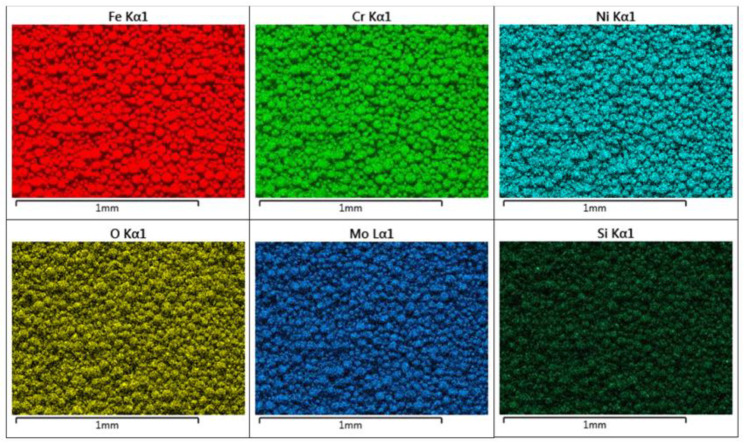
SEM EDX images showing elemental compositions for powder A. The elemental distribution appears very homogeneous.

Figure 9 shows representative SEM images from Powder A. Highly spherical particles of a poly-dispersed particle size range can be seen. In Figure 10, the chemical composition of the particles is shown for Powder A, obtained from EDX measurements. A homogeneous composition was found for the particles.

The electron microscopy analysis confirmed that the majority of the particles observed are highly spherical and possess a strongly homogenous elemental composition (Figure 10) In Figure 9a,b, a poly-dispersion of particle sizes is seen, with Powder B particles being more irregularly shaped in nature. Some satellite particles were also observed. However, the size and shape of the particles shown in Figure 9 appear to correlate well with the Particle Size Distribution (PSD) data, HS Circularity, aspect ratio and Circular Equivalent Diameter (CED) values of the particles as calculated from the visual microscope images used for the shape analysis investigation (Figure 7 and Figure 8). Indeed, the HS Circularity value for Powder B is lower than for Powder A (0.94 vs. 0.97), indicating that the small sample sets in Figure 7 and Figure 8 are suitably representative of the bulk powder (averaged across 100–200 K particles).

Elemental composition of powder A, as analysed via SEM-EDX is summarised in Table 4. The aluminium content is attributed to the SEM sample stubs, otherwise the values are as expected.

### 3.4. Tensile Properties

**Figure 11:**
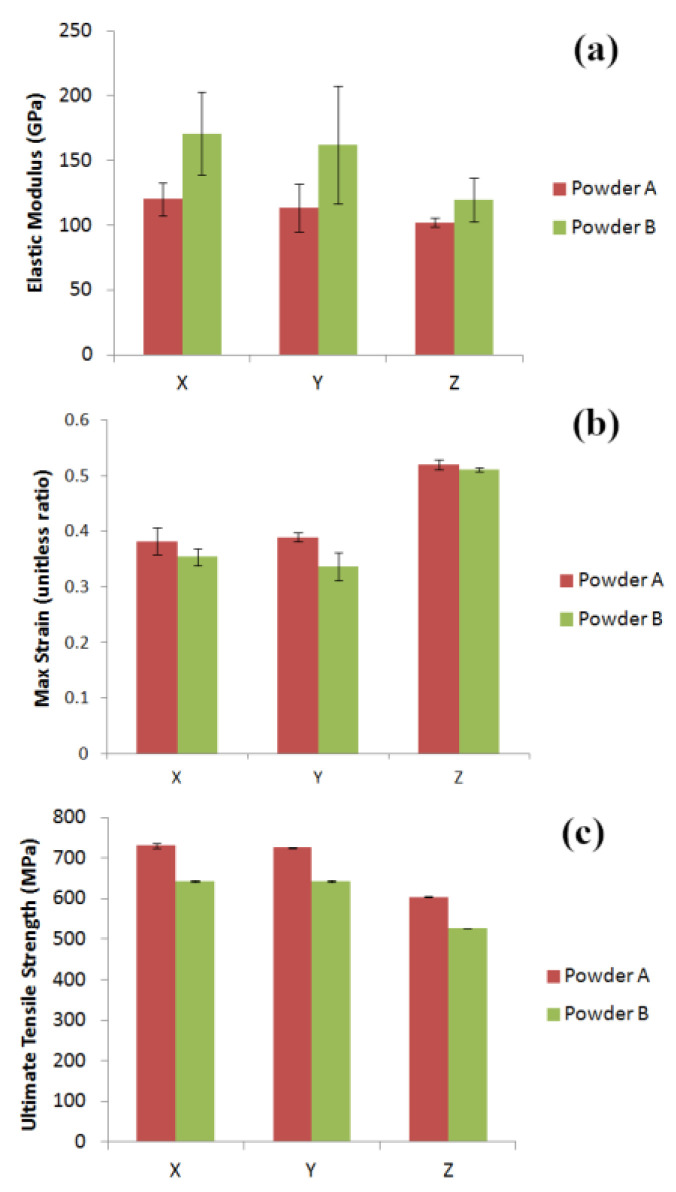
(**a**) Elastic moduli of parts produced using Powders A and B in X, Y and Z build orientations; (**b**) Max strain values exhibited by dogbone samples produced using Powders A and B as feedstock and (**c**) UTS values for samples from Powders A and B. Error bars are based on 95% confidence intervals.

In order to understand the interaction between feedstock properties and final part characteristics, the surface roughness, Ra, and the tensile properties of the parts were probed. Surface roughness measurements were performed before and after shot peening of the samples. Comparisons of elastic moduli of parts from Powder A and Powder B are illustrated in Figure 11.

The average as-printed surface roughness for the samples was found to range from 3.84–18.76 µm Ra for the different areas examined. Samples produced using Powder A typically gave parts with higher as-manufactured roughness values, with an average value of 12.7 µm, with a 95% confidence interval of 1.8 µm. The difference in as-printed roughness was found to be significant, with a *p*-value of 0.00573. This effect may be caused by the higher average particle size of Powder A (36.5 µm) than for Powder B (34.2 µm), for the D_50_ values. The variation in D_90_ is even more significant (65.4 vs. 51 µm) however the influence of this is not as pronounced judging from the Pearson calculation.

**Figure 12:**
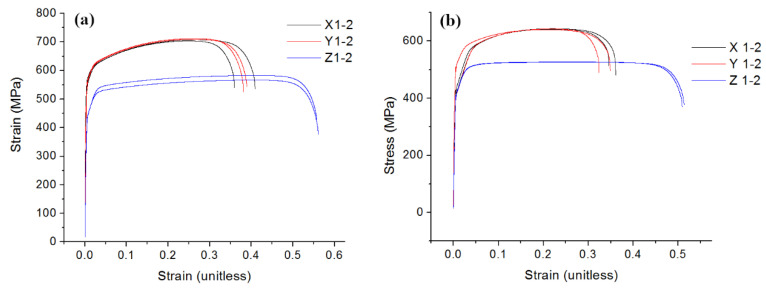
Stress–strain curves for the tensile samples produced via L-PBF using (**a**) Powder A and (**b**) Powder B, as feedstocks.

The UTS values for samples from both powders are higher for the samples built in the X and Y plane than those of the samples built vertically along the Z–axis. Samples from Powder A have higher UTS values in all build orientations (Figure 11 and Figure 12), while the samples from Powder B exhibited higher elastic strain. Max strain values are found for the samples built in the vertical axis for both powders. Figure 12 shows the stress–strain curves for the tensile samples produced using Powders A and B.

### 3.5. Effect of Powder Properties on Part Quality—A Pearson Correlation Study

A Pearson correlation was used to determine the level of dependency of the powder rheology on the shape and size of the particles. Data used for the correlation were taken from Table 2 and Table 3 and summarised in Table 5. The results of the correlation calculation are tabulated in Table 6. The Pearson correlation in Table 6 shows that powder rheological values for the powders are highly dependent on the particle size and shape.

Table 7 contains the Pearson correlations found for R_a_, UTS, max extension, modulus and particle size. The Pearson correlation coefficients showed strong positive correlation (ρ = 0.803) between the D_50_ of the particle size distribution and surface roughness before shot-peening. After shot peening, the average surface roughness measured for the same areas on the build plates ranged from 2.7–10.9 µm. The shot peened finish for the samples were found to not be significantly different, with a *p*-value of 0.945.

The modulus for samples produced oriented in the X-direction, the ultimate tensile strength for all build-orientations, and the maximum extensions for samples produced in the Y- and Z-directions showed significant variation, with *p*-values below 0.05.

For the average elastic modulus, a strong positive correlation was observed (ρ of 0.963 for the X orientated samples) with D_10_ values, indicating the importance of a well packed powder bed. For the UTS, a strong negative correlation was observed (ρ of −0.937, −0.971 and −0.896 for the X, Y and Z orientated samples, respectively) with D_10_. Conversely a strong positive correlation was found between D_50_ and D_90_ and UTS. For max extension values, a strong correlation between these and the D_90_, while a weak correlation was seen for the D_90_ values in the Z orientation. Max extensions for parts build in both Y and Z orientations exhibited moderate, diametrically opposed correlations to D_50_. Max extension for both Y and Z appear to be strongly negatively correlated to D_10_ values.

### 3.6. Micro-Sectional Analysis of Additively Manufactured Parts

**Figure 13:**
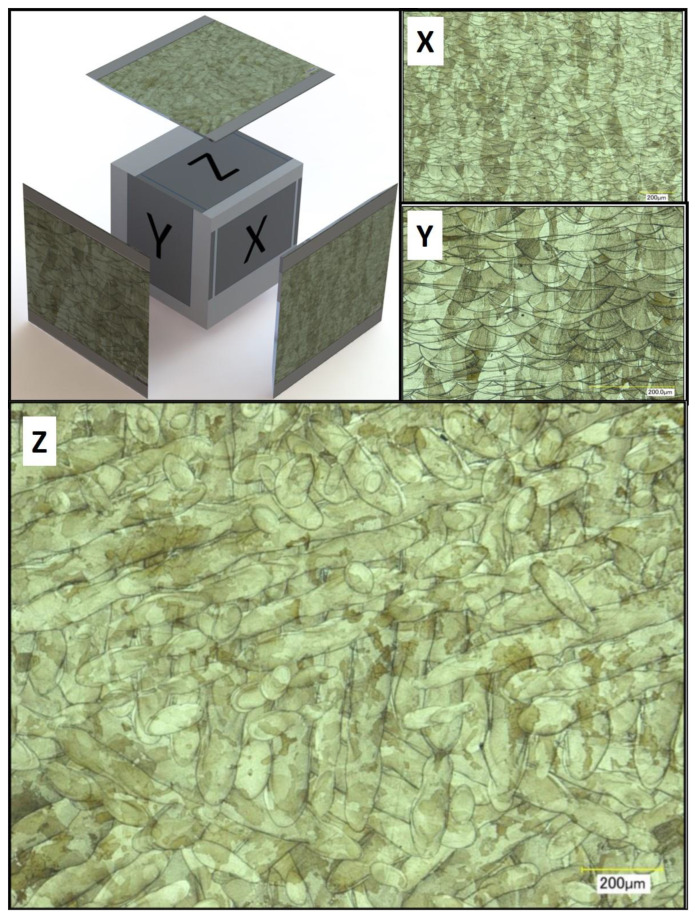
Illustration of micro-sections of cube printed with Powder A. All scale bars are 200 μm. **X**, **Y** and **Z** refer to the faces of the cube in relation to the melt pool (**Z**).

**Figure 14:**
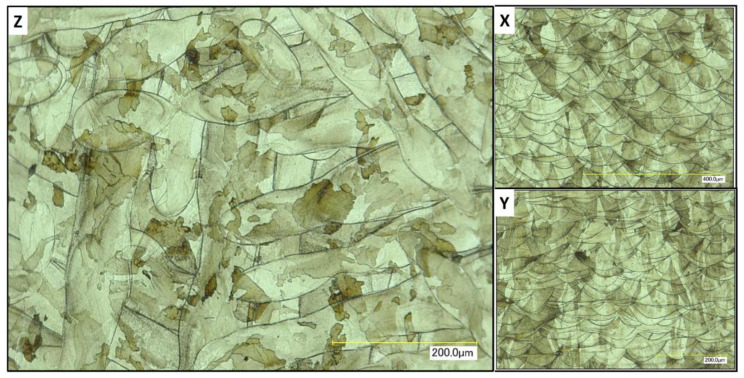
Illustration of micro-sections of cube printed with Powder B. Scale bars are (**X**) 400 μm, (**Y**) and (**Z**) 200 μm. **X**, **Y** and **Z** refer to the faces of the cube in relation to the melt pool (**Z**).

A strong correlation between powder rheology and powder shape and size has been established. In order to examine how important these factors are for the quality of a part fabricated in PBF, sample cubes of 5 by 5 by 5 mm were fabricated and microstructures examined. Figure 13 shows images of the micro-sections using Powder A from such a cube sample. Each face of the cube was imaged; X and Y were perpendicular to the melt pool face (Z). In Figure 14, good quality melt tracks are seen in the Z face, and consistent melt pools are seen in X and Y from the L-PBF process using powder B. These images suggest that not only do the powders possess good rheology, their spherical nature and good packing characteristics result in consistent melt pool formations. There appears to be a slight difference in the depth of the melt pool formation for Powder B, in the “X” and “Y” faces. However, both cube samples exhibit consistent melt pool tracks.

### 3.7. Fractography of Additively Manufactured Parts

In order to examine the effect of process and feedstock parameters on the mechanical properties, the fracture surfaces were imaged and compared with the recorded tensile results and porosity values. A similar approach has been used by Bidulský et al. [46] and Casati et al. [47] using electron microscopy. Table 8 shows the images and tensile values for the samples produced from powder A and B, measured in both the X and Z directions. The X and Y orientation samples exhibited a shear break with higher UTS values, while the Z orientation had a more complex macroscopic fracture morphology, more necking and fracture lip formation. It can be noted on a visual inspection of the samples, that all exhibited some degree of ductility with the larger degree of necking and lip formation on the Z produced samples relating to their recorded lower levels of UTS and higher levels of ductility.

Inset images of the fracture surfaces under higher magnification (300–500×) are also shown in Table 8, upon examination at higher magnification, no significant differences were observed at this increased scale, compared with the lower magnification (70–100×) images. Densities of the four selected tensile samples as measured by helium pycnometry ranged between 7.859 to 7.878 g/mL and exhibited very low variation. The densities measured are similar to those expected for 316L and do not indicate significant porosity, either from the L-PBF process or from the tensile test.

## 4. Discussion and Conclusions

A strong correlation between particle size and shape of Powders A and B with the rheological properties of the powders has been demonstrated. The rheological data show that both powders perform in a similar manner and are stable, flow well and fluidize easily. Both powders exhibit low cohesivity, with the difference in SE values between the two powders being directly attributed to the more irregular shape of powder B particles (aspect ratio and HS Circularity). This behaviour is expected given the high circularity and sphericity observed for the powders tested (shown in Figure 7 and Figure 8) and is considered to be a necessary property for a powder in additive manufacturing applications. The particle size data show poly-dispersed powders, with D_50_ values of around 33.8 ± 1.4 μm for powder A and 33.4 ± 5.4 μm for powder B.

The presence of fines (<10 µm) is typically expected to reduce flowability, due to higher static friction between particles. Since the particle size distribution of the powders tested in this work do not contain significant levels of fines, this effect has not been observed, however, Powder A did appear to have more than Powder B. The aeration test (Figure 4) suggests that both Powder A and B are easily fluidized (BFE values approaching zero at low air flow velocity), with Powder A exhibiting almost complete fluidization at 4 mm/s and Powder B completely fluidizing at 6 mm/s. Since the air flow required for the minimum (or zero) BFE to be reached is a direct indication of how easily fluidized a powder is, this has consequences where a high flow rate of inert gas is used within the L-PBF process chamber.

The presence of a larger number of fine particles in Powder A does not appear to adversely affect the rheological properties, indeed a slightly higher compressibility % value was observed, indicating a more efficient packing of the powder particles during the compressibility test. Powders A and B both show a homogeneous elemental composition, but while Powder A particles are highly spherical, Powder B contained more irregularly shaped particles, evidenced by the lower HS Circularity values, across their respective particle size ranges. In conclusion, Powder A possessed better rheological properties and was more spherical than Powder B, across the particle size range examined.

Both Powders A and B exhibit insensitivity to compression. While the absolute values are different; the trend across the increasing applied stress is the same. This is expected, given the highly dense nature of both powders. Compressibility of the powders is relevant in an AM tool where the powder layer is deposited by means of a rolling and compaction process. It can be concluded that the bulk-based approach of the FT4 instrument is more applicable to the understanding of powder mixing during deposition, transport of powder through the L-PBF machine and flow of powder during sintering or recoating.

A strong quantitative correlation exists between the rheological properties of the powders and their size and shape. The correlation suggests that more spherical powder particles have a lower BFE/g value and that the presence of fines appears to improve the flow–contrary to what some other researchers have found.

However, we found that using a layer height of 20 µm reduced the effect of smaller particles on UTS. It is well known from various manufacturing processes that grain orientation has a significant effect on UTS results [12,47,48]. This is known to be the case also for L-PBF and was found to be the case here also. The maximum extension was found to be strongly dependant on build orientation, and particle size appears to also play a strong role in this, particularly in the Y orientation, which again is likely due to the lower layer height selected, relative to the particle size distributions of the powders.

These results strongly indicate the suitability of both powders for use in additive manufacturing as evidenced by the consistent melt pool images and lack of porosity observed from the microstructure analysis (Figure 13 and Figure 14).

The results are summarised as follows:

1. There is a strong correlation between shape of particles and their size distribution, with their rheological properties.
2. Both powders exhibited acceptable rheological properties for L-PBF application.
3. A strong correlation between layer height, particle size and build orientation with resultant UTS and maximum elongation was determined with significantly higher maximum elongation measured in XY compared with Z orientation.
4. Both powders processed under the chosen L-PBF parameters gave acceptable microstructures.
5. The as-built roughness had a strong correlation with the D_50_ of the particle size distribution, with finer powders giving a smoother finish (Powder B).

## Figures and Tables

**Table 1:** Process parameters used in L-PBF processing of Powders A and B.

Parameter Name	Value
Ambient Temp	80 °C
Build Platform Temp	80 °C
O_2_ Chamber Concentration	0.1%
Recoater blade material	Stainless Steel
Laser Power	195 W
Beam Diameter	100 µm
Scan Velocity	1000–1200 mm/s
Layer Thickness	20 µm
Hatch Spacing	0.09 mm
Rotation Between Layers	67°
Strip Width	5 mm
Strip Overlap	0.12 mm
Laser Wavelength	1060–1100 nm

**Table 2:** Summary of particle size data for both powder A and B.

Average	Powder A	Powder B
**D_10_ (μm)**	15	23
**D_50_ (μm)**	36	33
**D_90_ (μm)**	65	50

**Table 3:** Calculated morphological parameters for Powders A and B.

Parameter	HS Circularity	Convexity	Solidity	Aspect Ratio
**Powder A**	0.97	0.998	0.997	0.926
**Powder B**	0.942	0.995	0.991	0.875

**Table 4:** Elemental composition of powder A measured by EDX. Values are expressed as counts-per-second/electronvolt (cps/eV).

Al	Si	Cr	Mn	Fe	Ni	Mo
0.29	0.51	18.66	1.90	67.18	9.18	2.27

**Table 5:** Rheological data used for Pearson correlation calculation examining the dependency of rheological data on particle size and shape for powders A and B.

Parameter	BFE (mJ)	SI	FRI	SE (mJ/g)	CBD (g/mL)
Powder A	620.14	1.11	1.31	1.84	5.12
Powder B	896.82	1.02	1.22	2.58	4.43

**Table 6:** Pearson correlation calculation examining the dependency of rheological data on particle shape for powders A and B.

Pearson Correlation (Size and Shape vs. Rheology)		
PEARSON (Powder A)	0.99	Dependent
PEARSON (Powder B)	0.99	Dependent

**Table 7:** Pearson correlation calculation examining the dependency of surface roughness and tensile strength on powder particle size.

Pearson Coefficients	D_10_	D_50_	D_90_
R_a_ before shot peen	0.357	0.803	0.35
X Build Orientation Modulus	0.963	−0.52	−0.901
Y Build Orientation Max Extension	−0.99	0.405	0.838
Z Build Orientation Max Extension	−0.707	−0.49	0.064
X Build Orientation UTS	−0.937	0.59	0.934
Y Build Orientation UTS	−0.971	0.492	0.887
Z Build Orientation UTS	−0.896	0.67	0.966

**Table 8:** Micrographs of tensile sample fracture surfaces. Scale bars for inset images are 200 µm for Powder A–X1 and Z1, Powder B–X2 and 500 µm for Powder B–Z1. Density values calculated using a Helium based Accupyc 1330 pycnometer. Inset images captured using a Keyence VHX2000 3D microscope (Keyence UK Ltd., Milton Keynes, UK).

Sample ID	Micrograph	UTS (MPa)	% Elongation	Density (kg/m^3^)
Powder A–X1	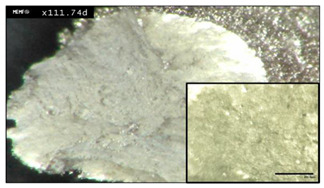	736	40	7859.7 St.Dev. 4.6/
Powder A–Z1	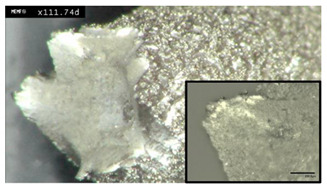	605	52	7859.2 St.Dev. 1.6/
Powder B–X2	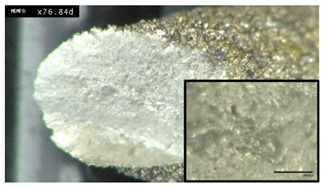	643	36	7878.00 St. Dev. 2.6/
Powder B–Z1	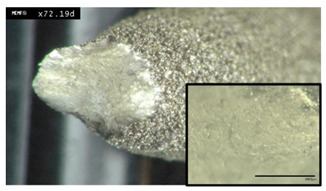	527	51	7876.2 St. Dev. 4/
